# Supplementary material for: Discovery and pharmacophoric characterization of chemokine network inhibitors using phage-display, saturation mutagenesis and computational modelling
Source: Nat Commun. 2023 Sep 16;14:5763. doi: 10.1038/s41467-023-41488-z (PMC10505172; doi:10.1038/s41467-023-41488-z)
Supplement: Supplementary file 4 — Source Data [file 41467_2023_41488_MOESM4_ESM.zip › SourceData/scripts/xNNKs_20230808183658.tar!/xNNKs/analysis/heatmaps/SV_HD2X_hydrophobic_heatmap.pdf]

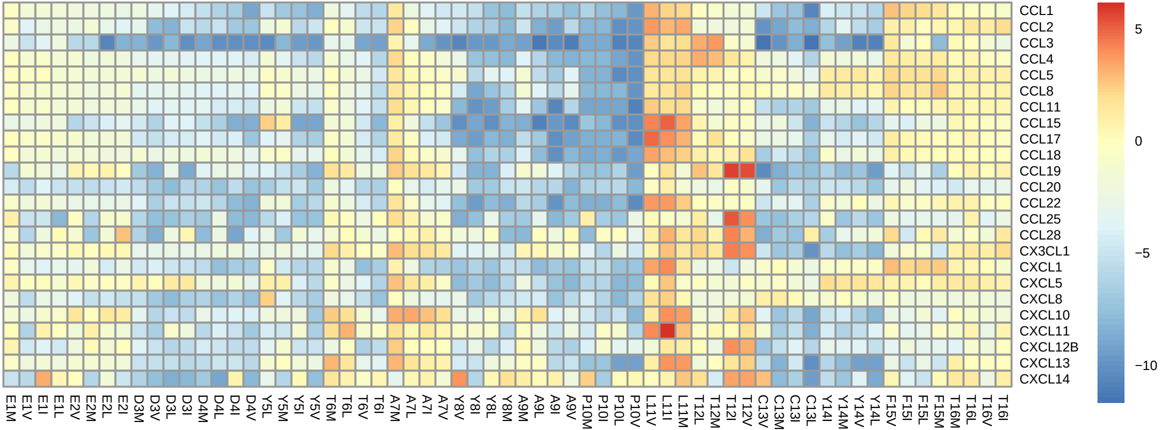

CCL1
CCL2
CCL3
CCL4
CCL5
CCL8
CCL11
CCL15
CCL17
CCL18
CCL19
CCL20
CCL22
CCL25
CCL28
CX3CL1
CXCL1
CXCL5
CXCL8
CXCL10
CXCL11
CXCL12B
CXCL13
CXCL14
5
0
−5
−10
E1M E1V E1I E1L E2V E2M E2L E2I D3M D3V D3L D3I D4M D4L D4I D4V Y5L Y5M Y5I Y5V T6M T6L T6V T6I A7M A7L A7I A7V Y8V Y8I Y8L Y8M A9M A9L A9I A9V P10M P10I P10L P10V L11V L11I L11M T12L T12M T12I T12V C13V C13M C13I C13L Y14I Y14M Y14V Y14L F15V F15I F15L F15M T16M T16L T16V T16I
